# Reliability of MR-Based Volumetric 3-D Analysis of Pelvic Muscles among Subjects with Low Back with Leg Pain and Healthy Volunteers

**DOI:** 10.1371/journal.pone.0159587

**Published:** 2016-07-26

**Authors:** Elżbieta Skorupska, Przemysław Keczmer, Rafał M. Łochowski, Paulina Tomal, Michał Rychlik, Włodzimierz Samborski

**Affiliations:** 1 Department of Rheumatology and Rehabilitation, Poznan University of Medical Sciences, Poznan, Poland; 2 Department of Mathematics and Mathematical Economics, Warsaw School of Economics, Warsaw, Poland; 3 Department of Virtual Engineering, Poznan University of Technology, Poznan, Poland; Georgia Regents University, UNITED STATES

## Abstract

**Aim:**

Lately, the diagnostic value of magnetic resonance imaging, Lasègue sign and classic neurological signs have been considered not accurate enough to distinguish the radicular from non-radicular low back with leg pain (LBLP) and a calculation of the symptomatic side muscle volume has been indicated as a probable valuable marker. However, only the multifidus muscle volume has been calculated so far. The main objective of the study was to verify whether LBLP subjects presented symptomatic side pelvic muscle atrophy compared to healthy volunteers. The second aim was to assess the inter-rater reliability of 3-D manual method for segmenting and measuring the volume of the gluteus maximus, gluteus medius, gluteus minimus and piriformis muscles in both LBLP patients and healthy subjects.

**Method:**

Two independent raters analyzed MR images of LBLP and healthy subjects towards muscle volume of four pelvic muscles, i.e. the piriformis, gluteus minimus, gluteus medius and gluteus maximus. For both sides, the MR images of the muscles without adipose tissue infiltration were manually segmented in 3-D medical images.

**Results:**

Symptomatic muscle atrophy was confirmed in only over 50% of LBLP subjects (gluteus maximus (p<0.001), gluteus minimus (p<0.01) and piriformis (p<0.05)). The ICC values indicated that the inter-rater reproducibility was greater than 0.90 for all measurements (LBLP and healthy subjects), except for the measurement of the right gluteus medius muscle in LBLP patients, which was equal to 0.848.

**Conclusion:**

More than 50% of LBLP subjects presented symptomatic gluteus maximus, gluteus minimus and piriformis muscle atrophy. 3-D manual segmentation reliably measured muscle volume in all the measured pelvic muscles in both healthy and LBLP subjects. To answer the question of what kind of muscle atrophy is indicative of radicular or non-radicular pain further studies are required.

## Introduction

Low back with leg pain patients have been defined in diverse ways, from those with any leg pain to those with radiculopathy and an MRI-confirmed clinical diagnosis of nerve root compression [1]. Lumbosacral radicular syndrome (LSRS) defined as sciatica or sciatic neuralgia is frequently diagnosed with a yearly incidence of 5–10 per 1,000 persons [2]. The diagnosis of LSRS is based on the neurological bedside examination, magnetic resonance imagining (MRI) and Lasègue sign interpretation. However, it is impossible to distinguish LSRS from atypical leg pain (also called pseudoradicular), motion-segment or facet joint pain [3,4]. Moreover, the diagnostic value of MRI, Lasègue sign and classic neurological signs have recently been proven not as accurate as it was assumed before [5–9].

Lastly, Kader et al. proposed that MRI assessment towards muscle atrophy may provide some extra information which can facilitate the diagnosis of low back with leg pain (LBLP) patients [10]. Only few studies concerning symptomatic muscle atrophy in patients with lumbosacral radiculopathy are available. Additionally, all of them focus on the multifidus (MF) muscle [10–14].

However, the MF muscle seems to be of little importance when it comes to diagnosing LBLP subjects. Symptomatic MF muscle atrophy is well documented in low back pain (LBP) patients and it would be difficult to assess whether MF atrophy is due to LBP or LBLP. The role of paraspinal muscles in the causation of LBP and sciatica remains unclear. Hyun et al. speculated that asymmetric MF atrophy observed in patients with unilateral lumbosacral radiculopathy with disc herniation was a result of a denervation process [14], but Kader et al. reported bilateral and multilevel MF degeneration even in patients with a single nerve root irritation [10]. Another hypothesis is related to reflex inhibition, which is about reducing alpha motoneurons activity in the anterior horns of the spinal cord, resulting in muscle activity suppression [15]. Among LBP patients, the unisegmental MF innervation [16,17] can result in symptomatic atrophy limited to one vertebral level. Thus, the confirmation of atrophy in other muscles related to different subtypes of LBLP could have a possible differential diagnostic value [18–24].

It seems that muscle volume (MV) calculation of pelvic muscles can provide some additional information. The weakness of the gluteus medius muscle in pregnant women with psuedoradicular leg pain [25,26], as well as the gluteus maximus, gluteus minimus and piriformis muscle alterations on the symptomatic side in subjects with sciatic or sciatic-like pain, have been confirmed [27,28].

There are a few studies on MV calculation of small pelvic muscles in pain states [29–31]. To date, however, no study has reported pelvic muscle volume among LBLP subjects. Based on Kader et al.’s [10] study and the confirmation of similar MV of dominant and non-dominant side (pelvis and lower extremities muscles) in healthy humans [32], we hypothesize that some chronic LBLP subjects could present pelvic MV decrease on the symptomatic side.

The muscle volume measurement using the MRI picture is commonly considered a reliable technique. Although MV of some pelvic muscles has been reported by a few authors [30,33,34], a reliable distinction between gluteus medius and gluteus minimus can be difficult due to their anatomical variability. Furthermore, normative MV values for pelvic muscles are lacking [30,35].

The present study was designed to determine the most accurate results under given circumstances. Firstly, a 3-D muscle volume calculation rather than the most commonly used single cross-sectional area (CSA) measurement was performed. The CSA measurement is faster but not always appropriate for muscle volume. What is more, the optimal level to carry out the measurement is difficult to define and then reproduce [36–39]. Secondly, the manual technique instead of an automated or a semi-automated method was employed to avoid the difficulty in differentiating between the gluteus minimus and medius muscles.

Thus, the reliability of using the 3-D method with regard to a specific pelvic muscle has to be measured before side-to-side percentage difference calculation of this single muscle is performed. Furthermore, to check the importance of the volume measurement of pelvic muscles as a diagnostic tool for LBLP patients, the symptomatic incidence of probable pelvic muscles atrophy would have to be established first. It is possible that not every LBLP patient presents symptomatic muscle atrophy due to a complicated pain pathomechanism. A recent MR imaging study showed that 40% out of 126 men who were asymptomatic had multifidus muscle asymmetry exceeding 10%, therefore caution in using level- and side-specific paraspinal muscle asymmetry for identifying subjects with LBP and spinal pathology was advised [40].

As soon as the reliability of this method is confirmed, the next step for the future study should be to analyze the symptomatic MV, followed by checking specific muscles against different types of LBLP, e.g. muscle pain, sacroiliac joint syndrome, etc.

The main objective of the study was to verify whether LBLP subjects presented symptomatic side pelvic muscle atrophy compared to healthy volunteers. The second aim was to assess the inter-rater reliability of 3-D manual method for segmenting and measuring the volume of the gluteus maximus (GMax), gluteus medius (GMed), gluteus minimus (GMin) and piriformis (Pir) muscles in LBLP and healthy subjects.

## Material and Methods

Seventy one MR images of subjects with low back with leg pain and twenty nine of healthy volunteers were used in the study. Two independent raters manually segmented 3-D MR images of four pelvic muscles: the piriformis, gluteus minimus, gluteus medius and gluteus maximus (both sides). The muscles were segmented without adipose tissue infiltration. The Poznan University Institutional Review Board approved the study protocol no. 574/14. The MR images were obtained in an ongoing study involving low back with leg pain subjects (27 men, 44 women; mean age 47.7 ± 8.4) and healthy volunteers (10 men, 19 women; mean age 47.6 ± 9.9). Table 1 shows a clinical description of the group whose muscle volume was measured. Written informed consents were obtained from the subjects before enrollment. The inclusion criteria for low back with leg pain subjects were as follows: current MRI, positive Lasègue sign, subacute or chronic pain state, age between 30 and 60 (inclusive), both lower limbs present, >3 on the 1–10 point VAS scale of leg pain, with this being the dominant pain problem. The inclusion criteria for healthy volunteers were: general good health, age between 30–60 (inclusive), both lower limbs present. The key exclusion criteria for all subjects were: complex regional pain syndrome, cauda equina syndrome, previous back surgery, spinal tumors, scoliosis, pregnancy, coagulant treatment, disseminated intravascular coagulation, diabetes, epilepsy, infection, inflammatory rheumatologic diseases, stroke or oncological history.

**Table 1:** Clinical description of the analyzed group.

	MRI [%]						Neurological examination [%]							
Participants	Disc prolapse			Root compression			Sensory deficits				Lasèque sign		Tendon reflex	
													Patellar	Ankle
	L_3-4_	L_4-5_	L_5_-S_1_	L_4_	L_5_	S_1_	L_3_	L_4_	L_5_	S_1_	>45	<45	Absence	Absence
LBLP	26.76	64.79	54.93	26.76	29.58	22.54	14.08	15.49	26.76	19.72	53.52	46.48	4.23	22.54
Healthy volunteers	13.78	44.83	41.38	17.24	20.69	6.90	0.00	0.00	0.00	0.00	-	-	3.45	6.90
p-value *	0.256	0.106	0.313	0.451	0.501	0.119	-	-	-	-	-	-	-	0.119

p–the Chi^2^

### Magnetic resonance imaging: image acquisition

A 1.5 Tesla Signa HDe system (GE) scanner was used to acquire the images. The images of each subject were taken bilaterally and covered the area from the lumbar spine down to pelvic and upper thigh muscles to allow the assessment of the pelvic muscles. Sagittal T2-weighted images were acquired according to an MR imaging protocol consisting of a pulse sequence (repetition time (TR): 3.500, echo time (TE): 110, field of view (FOV): 100%, matrix: 320 x 224), using a slice thickness of 4mm without an interslice space. The MR images were obtained with subjects lying supine.

A manual segmentation requires an acquisition of contiguous axial anatomical cross-sectional images, followed by offline analyses of each serial image, using contour tracing of each individual muscle of interest [41]. Images were recorded with the aid of DICOM (Digital Imaging and Communications in Medicine) used for distributing medical images regardless of using the scanner. The lumbo-pelvic MR images (DICOM format) were converted to NIfTI format to enable the analysis in the ITK-SNAP 2.2.0 software program. ITK-SNAP is a free open-source software application used to segment structures in 3-D medical images [42–44].

### Data analysis: image segmentation

For both sides, volumetric images of four muscles, i.e. the piriformis, gluteus minimus, gluteus medius and gluteus maximus were acquired. Adipose tissue infiltration was not taken into account.

**Fig 1:**
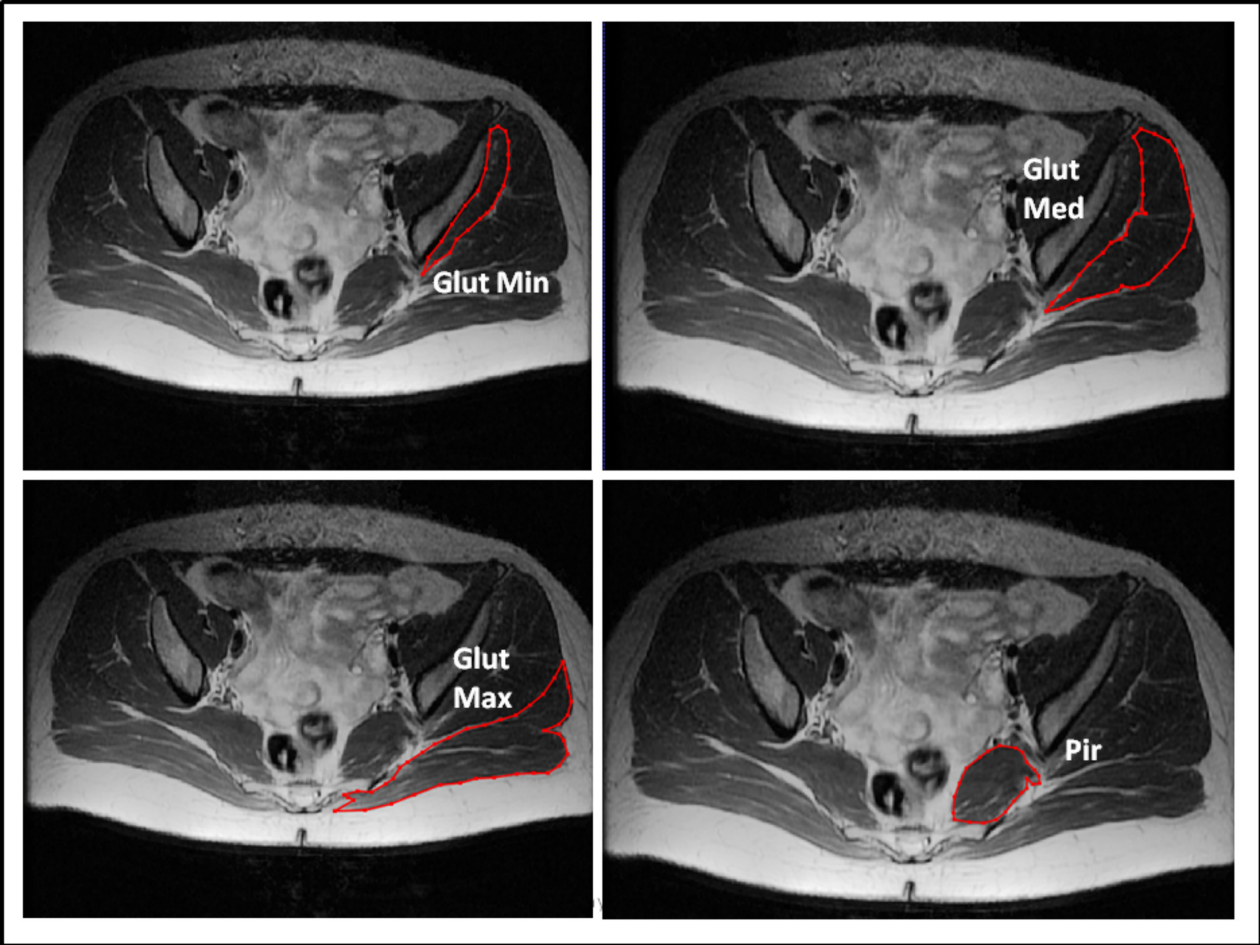
Samples of a single MRI slice with contouring of the four segmented muscles (axial view). Legend: Glut min–gluteus minimus muscle, Glut med–gluteus medius muscle, Glut max–gluteus maximus muscle, Pir–piriformis muscle. Muscles were contoured in ITK-SNAP where the structures were outlined slice-by-slice by pointing and clicking with a mouse. The program connected the consecutive points with lines.

**Fig 2:**
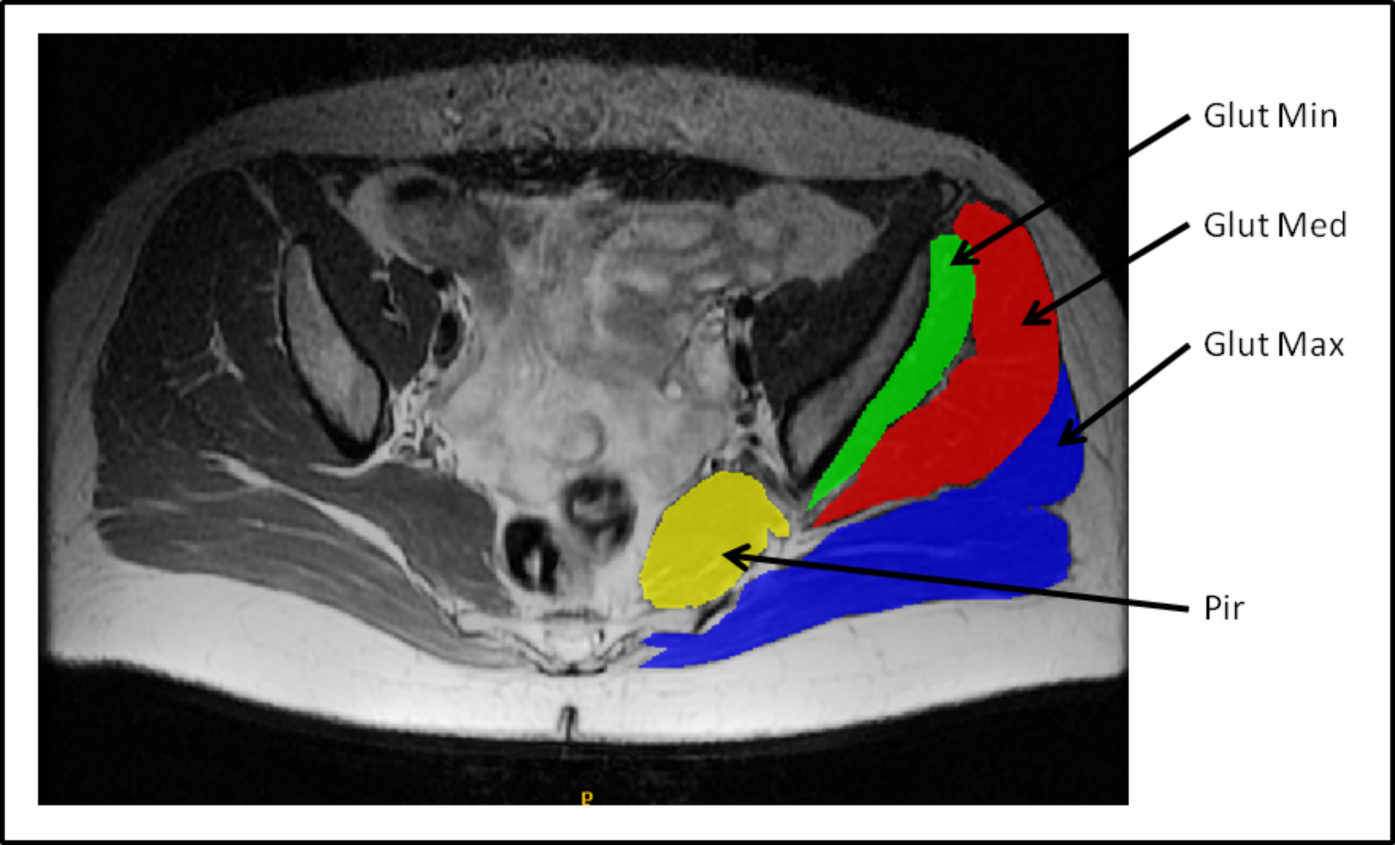
Sample of a single MRI slice with final segmentation of the four chosen muscles. Legend: Glut min–gluteus minimus muscle, Glut med–gluteus medius muscle, Glut max–gluteus maximus muscle, Pir–piriformis muscle. All anatomical objects defined by closed contours were filled in by selected colors.

Image segmentation refers to the process of outlining the shape of structures visible in the cross-sections of a volumetric data set. The segmentation was made from the origin to the insertion of each muscle. During the segmentation, the borders of each muscle were clearly identified. Various muscle slices were segmented: from 55 to 60 images of the gluteus maximus, 40 to 45 images of the gluteus medius, 30 to 35 images of the gluteus minimus and 15 to 20 images of the piriformis. Variations in the number of MR images were due to the differences in the lumbo-pelvic area size of the specimens. Subcutaneous adipose tissue (SAT) and intermuscular adipose tissue (IMAT) volumes were excluded from the calculation. The infiltrated adipose tissue appeared as a T1-high-intensity signal in comparison with the muscles [45]. In the present study, IMAT interspersed between the muscles was differentiated from SAT by drawing a line along the deep fascial plane surrounding a given muscle [46]. The manual mode, i.e. hand contouring, was applied for segmentation. The structures were outlined slice-by-slice by pointing and clicking with a mouse. The program connected the consecutive points with lines (Fig 1). All anatomical objects were defined by closed contours which were filled in by selected colors (Fig 2). The volume of each side was determined and calculated automatically by adding the number of voxels contained in the muscle and multiplying the sum by voxel dimension (mm^3^).

#### Inter-rater measurement

Two physical therapists, who are also engaged in a full-time clinical research, served as raters. One of them (PK, rater 1) has approximately three years of experience in segmenting individual muscles using ITK-SNAP. The other physical therapist (PT, rater 2) had no prior experience in using the ITK-SNAP segmentation tool. The raters met three times with radiologists to establish standardized approaches to identifying and separating fat and muscle borders. Additionally, each rater received approximately 5 days of training from a physician experienced in radiology segmentation to be able to determine how ITK-SNAP and each muscle compartment are defined. Then, the raters practised how to use the ITK-SNAP program to identify and measure muscle volume based on a few training LBLP cases not included in the study. During data acquisition, rater 1 was only practising the pelvic muscle segmentation on cases not included in the study. All data was analyzed for a few consecutive days.

#### Sciatic impact of muscle size

Similar pelvic muscle volume for dominant and non-dominant sides in both genders was confirmed among healthy volunteers [31]. Although a high incidence of symptomatic side muscle atrophy for LBP has been confirmed, not every LBP case presented that. Thus, due to a complicated pain pathomechanism (e.g. radicular, psuedoradicular, etc.), which can influence MV in different ways, the same situation would probably be observed among LBLP subjects. The event of interest was side-to-side comparison of muscle volume of healthy volunteers and then, the same for painful to non-painful side of LBLP subjects. The assumption for the LBLP group was to divide it into two groups: one with the left symptomatic side and the other with the right symptomatic side. It was established that the incidence of smaller muscle volume of the symptomatic side could be diagnostically valuable if significantly more than 50% of cases presented MV decrease on the symptomatic side. This assumption was based on findings from testing LBP patients for whom symptomatic MF muscle atrophy was estimated at around 80% maximum compared to maximum 40% among healthy volunteers [47].

### Statistical analysis

#### Impact of sciatic pain on muscle volume

The statistical analysis was conducted by a blind researcher for the compressive forces applied. To assess the impact of LBLP presence on the volume of the corresponding muscle, the exact binomial tests were conducted. The value of the muscle volume was presented as a mean and standard deviation (SD). Since the Shapiro-Wilk test rejected the normality of the muscle volume data for almost all muscles, the Wilcoxon signed rank test was used to assess significant side-to-side differences in the characteristics of the central tendency (mean) for both LBLP and healthy volunteers (right to left and symptomatic to non-symptomatic sides). A significance level of 5% was adopted to all analyses.

#### Test-retest measurement

To assess the reliability and consistency of muscle volume measurements, two similar statistics were used–the concordance correlation coefficient and the intra-class correlation coefficient between the measurements made by two independent raters (rater 1 and rater 2). The concordance correlation coefficient was calculated in the way proposed by Nickerson [48]. The intra-class correlation coefficient (ICC) was calculated as proposed by Fisher [49].

Intra-class correlation coefficients above 0.90 are usually considered excellent, values from 0.75 to 0.90 are considered good and below 0.75 –poor to moderate.

In all statistical analyses, we used the R free software environment for statistical computing and graphics, version 3.2.0.

## Results and Discussion

### Clinical meaning of pelvic muscle volume measurement

The exact binominal test has confirmed that more than 50% of LBLP patients presented a smaller volume for all pelvic muscles for the symptomatic side, both left and right, except for GMed (Table 2). The percentage side differences in the average muscle volume in LBLP patients for raters 1 and 2 were respectively: GMax (6.13% and 6.29%), GMed (2.98% and 3.69%), GMin (7.27% and 5.95%) and Pir (8.2% and 8.91%) and the Wilcoxon signed rank test confirmed significant differences for all muscles (p<0.001), except for GMed (p = 0.077) (Table 3).

**Table 2:** Differences in the frequency of LBLP patients with a smaller volume of the left muscles compared to the right muscles.

			LBLP patients			
Muscle	Painful side	Researcher	n = (Vol_left_<Vol_right_)	n = (Vol_left_<Vol_right_)	p-value	95% confidence interval
GMax	left	1	39	2	<0.001	(0.85, 1)
		2	37	4	<0.001	(0.79, 1)
	right	1	5	25	<0.001	(0, 0.32)
		2	5	25	<0.001	(0, 0.32)
GMed	left	1	26	14	0.040	(0.51, 1)
		2	25	15	0.077	(0.48, 1)
	right	1	14	17	0.360	(0, 0.61)
		2	13	18	0.237	(0, 0.58)
GMin	left	1	31	10	<0.001	(0.62, 1)
		2	28	13	0.014	(0.54, 1)
	right	1	4	26	<0.001	(0, 0.28)
		2	6	24	<0.001	(0, 0.36)
Pir	left	1	32	9	<0.001	(0.65, 1)
		2	33	8	<0.001	(0.68, 1)
	right	1	8	22	<0.001	(0, 0.43)
		2	10	20	0.049	(0, 0.50)

p–value of the exact binomial test

**Table 3:** Comparison of the mean values for the four pelvic muscles volume (symptomatic v. non-symptomatic side of the LBLP group).

Pelvic Muscle	Rater	Mean MV±SD		p value
		Symptomatic side	Non-symptomatic Side	
GMax	1	672997.7 ± 132687.9	715571±144140.4	_<0.001_
	2	668439.7±133899.4	711815±142749.7	_<0.001_
GMed	1	277369.7±45887.38	285759.2±52299.07	_0.077_
	2	276749.9±57113.54	287154.7±57775.21	_0.077_
GMin	1	74224.92±18035.65	79827.08±18697.04	_<0.001_
	2	74779.36±18026.08	79368.71±19401.51	_<0.001_
Pir	1	26565.83±7792.95	28836.94±8915.23	_<0.001_
	2	26879.36±7853.31	29386.14±8894.19	_<0.001_

p–value of the Wilcoxon signed rank test

For all pelvic muscles of healthy volunteers, the exact binominal tests did not reveal any significant differences in the frequency of subjects with a smaller volume of the left muscles compared to the right muscles (Table 4). The percentage side differences in the average muscle volume of healthy volunteers for raters 1 and 2 were respectively: GMax (0.79% and 1.24%), GMed (3% and 2.61%), GMin (0.61% and 0.49%) and Pir (0.42% and 0.16%) and the Wilcoxon signed rank test confirmed a lack of significant differences, except for GMed (p<0.05).

**Table 4:** Differences in the frequency of healthy volunteers with a smaller volume of the left muscles compared to the right muscles.

	Healthy volunteers				
Muscle	Rater	n = (Vol_left_<Vol_right_)	n = (Vol_left_<Vol_right_)	p-value	95% confidence interval
GMax	1	12	17	0.458	(0.24, 0.61)
	2	10	19	0.136	(0.18, 0.54)
GMed	1	8	21	0.024	(0.13, 0.47)
	2	9	20	0.061	(0.15, 0.51)
GMin	1	13	16	0.711	(0.26, 0.64)
	2	14	15	1	(0.29, 0.67)
Pir	1	12	17	0.458	(0.24, 0.61)
	2	12	17	0.458	(0.24, 0.61)

p–value of the exact binomial test

### Results of the reliability and consistency assessment of volume measurement of four pelvic muscles

**Fig 3:**
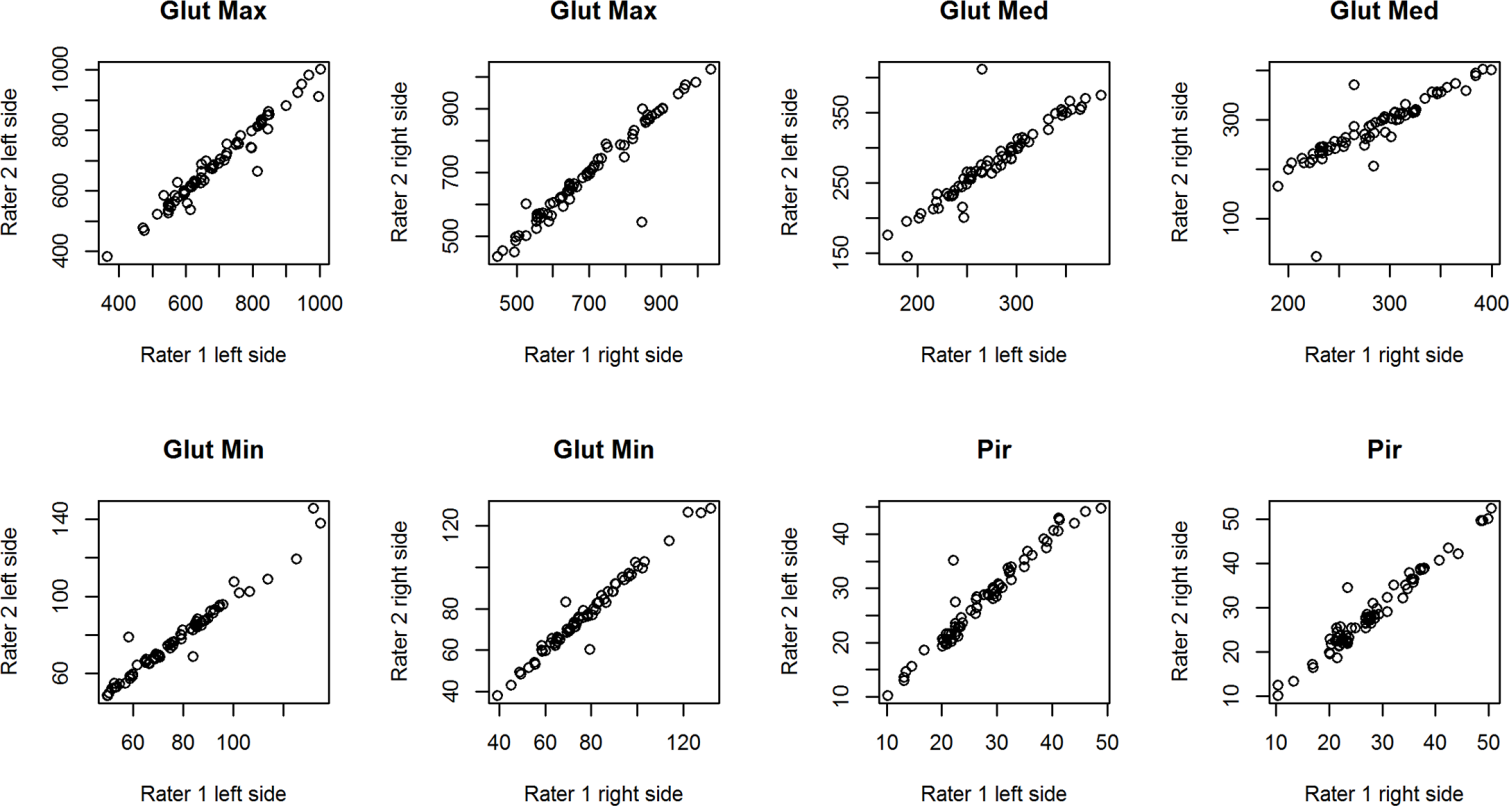
Overall scatter of volume measurements in [cm^3^] performed by two independent raters (1 and 2) in LBLP patients.

**Fig 4:**
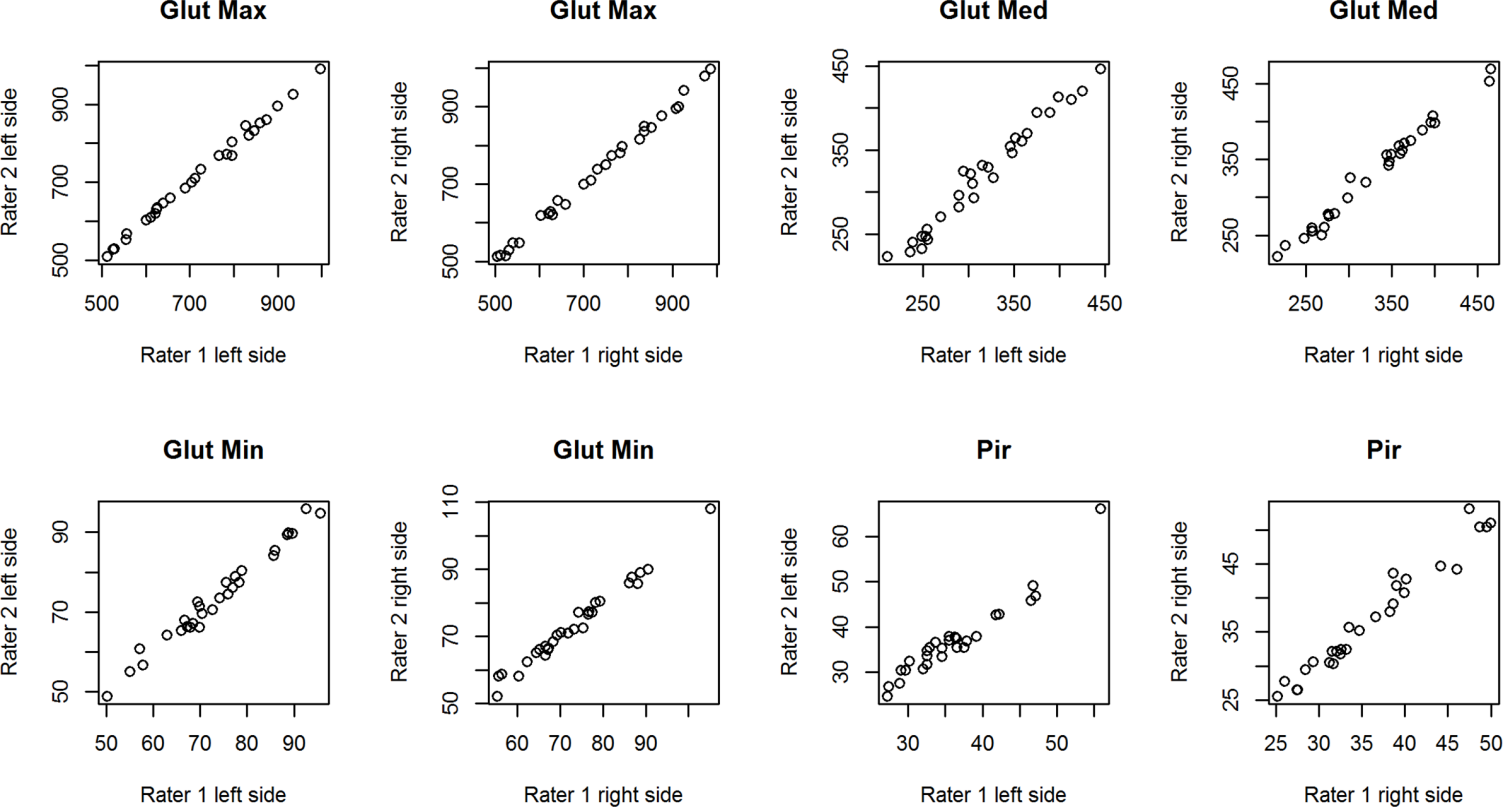
Overall scatter of volume measurements in [cm^3^] performed by two independent raters (1 and 2) in healthy volunteers.

Table 5 summarizes the assessment of the reliability and consistency of volume measurement of four pelvic muscles performed by two independent raters. The ICC values indicate that the inter-rater reproducibility was greater than 0.90 for all measurements, except for the measurement of the right gluteus medius muscle of LBLP patients which was equal to 0.848. Fig 3. presents the overall scatter of the data corresponding to the inter-rater agreement for each of the muscles in LBLP subjects. The plot shows a good distribution of data points around the 45° degree line, which indicates a high level of agreement between the raters. The same results were confirmed for healthy volunteers and are shown in Fig 4.

**Table 5:** Concordance and intra-class correlations between volume measurements made by two independent raters (1 and 2) in LBLP patients and healthy volunteers.

Muscle	Side	LBLP patients		Healthy volunteers	
		Concordance Correlation	ICC	Concordance Correlation	ICC
GMax	R	0.961	0.961	0.998	0.998
	L	0.976	0.976	0.997	0.997
GMed	R	0.848	0.848	0.993	0.992
	L	0.917	0.917	0.984	0.984
GMin	R	0.985	0.985	0.990	0.990
	L	0.978	0.978	0.989	0.989
Pir	R	0.975	0.975	0.971	0.970
	L	0.969	0.969	0.942	0.942

R–right; L–left; ICC–intra-class correlation

This is the first time when symptomatic pelvic muscle atrophy was calculated for LBLP patients and the results have confirmed that more than 50% of cases presented a significantly smaller muscle volume of the symptomatic side for GMax, GMin, Pir, except GMed (Tables 2 and 3). Until now, all available studies concerning the percentage incidence of symptomatic muscle atrophy were focused on the MF muscle, most commonly among LBP patients, and the atrophy was observed in 20–60% of cases [50,51]. More recently, when a particular spine level (L4–5 and L5–S1) was considered, the atrophy was confirmed in around 80% of subjects [10]. Kadar et al. confirmed a significant correlation between lumbar MF muscle atrophy and leg pain (radicular and non-radicular), but almost half of the group had MF muscle atrophy without any other MRI abnormalities such as herniated nucleus pulposus, spinal stenosis or nerve root compression [10]. This indicates that MF muscle atrophy was probably caused by different pathological changes. It is not known if the same would be true for pelvic muscles in LBLP subjects. It is crucial, though, to analyze every single pelvic MV while taking into account neurological examination results and/or co-diagnosis of specific states as muscle pain, sacroiliac joint syndrome, facet joint syndrome, etc. This would possibly enable the diagnosis of radicular and non-radicular component of LBLP and the results of such an analysis should be examined in further studies.

The results obtained for the control group were consistent with other studies, which confirmed some kind of MV atrophy [32] and the side-to-side comparison for all measured muscles revealed non-significant differences under 1.24%, except for GMed (3% and 2.61%; p<0.05). Interestingly, Marcon et al. reported the normative value of GMed and GMin muscle volume and they confirmed no age, gender and dominant leg dependency [52]. It is not known what could possibly influence the GMed results of the control group. Further studies concerning the reliability of MV measurement for GMed and GMin of healthy subjects are necessary.

#### Hypothetical explanation of observed symptomatic pelvic muscle atrophy

One of possible explanations of symptomatic pelvic muscle atrophy observed in the present study can be neurogenic type atrophy due to nerve compression, which provoked metabolic changes in the sympathetic nervous system, then metabolic activity of the musculoskeletal system, vasoconstriction and, finally, atrophy [53]. That kind of muscle atrophy can probably be observed among LBLP patients with neuropathic pain co-existence, which has been confirmed in around 30% of cases [4]. However, it is difficult to point out the muscle or muscles which are prone to atrophy. Additionally, a vasomotor reaction in the pain area has been confirmed for around 30% of cases of subacute and chronic sciatica with muscle pain co-existence [54,55]. However, they had no sign of persistent vasoconstriction and noxious stimulated vasodilatation was evoked for a short time. Thus, it is not clear if muscle atrophy can be actually observed or not. Nevertheless, in this subgroup of LBLP patients, a symptomatic MV decrease can be expected within e.g. the gluteus minimus and piriformis, which are indicated as origins of referred sciatic-like pain.

Another possible explanation of pelvic muscle atrophy among LBLP patients can be inactivity due to changes in balance between muscle fiber apoptosis and regeneration [56,57]. This type of muscle atrophy can be observed as a result of the patient sparing the symptomatic leg or due to an improper functioning of the muscles responsible for trunk and pelvis stability, which was indicated as one of the main contributors to chronic low back pain [27,58–62]. GMed was pointed out as a probable indicator of SIJ based on the assumption that it has the control function of femoral motion primarily during dynamic lower extremity motion and stabilization of the pelvis in the frontal and transverse planes [25,63,64]. In the present study, the side differences for GMed MV were similar in LBLP and healthy volunteers (around 3%). Surprisingly, significant results were confirmed only for the control group. If GMed atrophy proved to be indicative of the SIJ syndrome exclusively, it would be present in 15–30% of LBLP patients and the MV percentage difference would be much higher than 3% [65–71]. Moreover, it was postulated that the gluteus maximus together with the quadratus lumborum, rather than the gluteus medius, has a crucial meaning for lumbopelvic stabilization in subjects with the sacroiliac joint syndrome [72,73]. Based on the present study, it seems that significantly atrophic gluteus maximus can probably be valuable for the sacroiliac joint syndrome (Tables 2 and 3). However, it would be worth checking both GMax and GMed among LBLP subjects presenting SIJ. Further studies concerning the SIJ group exclusively could answer the question of which muscle would be indicative of pseudoradicular LBLP or not.

#### Reliability of 3-D manual segmentation for pelvic muscles

The results have confirmed that the 3-D manual segmentation reliably measured muscle volume in the gluteus minimus, gluteus medius, gluteus maximus and piriformis muscles in both healthy and LBLP subjects (Table 5; Figs 3 and 4). However, the moderate agreement for the right gluteus medius of the experimental group (Table 5) can be explained by the choice of the manual segmentation, which is burdened with a higher personal error compared to an automated or semi-automated method [74,75]. Nevertheless, automated methods are desirable when the contrast with the surrounding organs is high. Thus, due to the gluteus minimus and medius muscles’ anatomy and problems with separating these muscles, automated methods are of little use at this moment. Barnouin et al. described similar problems concerning the vastus lateralis and vastus intermedius manual segmentation [76]. It has been proposed to group two muscles together when the border between the muscles is not clear [50, 77]. However, the gluteus minimus and glutes medius separation seems to be essential for LBLP subjects, especially when the impact of muscle pain which fakes sciatic pain is considered. Similarly to Barnouin et al., the solution was to use the bean pattern for the gluteus minimus and medius muscles and visible landmarks of the previous and next images [76].

Another problem for a possible use of pelvic muscles as a specific radicular vs. non-radicular indicator is the time needed for analysis. 3-D manual segmentation is extremely costly, hence why the single cross-sectional area (CSA) measurement is most commonly used instead. Unfortunately, the CSA measurement is not always appropriate for MV and the optimal level to carry out the measurement is both difficult to define and reproduce [36–39]. It seems the CSA measurement is not useful as a diagnostic tool for pelvic muscles after all because of the previously discussed difficulty in separating the gluteus minimus from the gluteus medius. Nevertheless, it would be valuable to compare the reliability and size results of both 3-D muscle volume and CSA measurements performed on the same group of LBLP subjects, especially with pelvic muscle atrophy proposed as a diagnostic tool.

#### Limitations

The results of this study are limited due to the lack of intra-rater reliability. As the manual segmentation was chosen as a more valuable method, a higher number of subjects was needed. Due to this study being extremely time-consuming, the inter-rater reliability analysis will have to be done in a separate study.

## Conclusions

The idea for the study was based on the assumption that MV calculation can provide some additional diagnostic information for radicular and non-radicular LBLP subjects. It seems that the piriformis, gluteus minimus and maximus MV measurement probably have some diagnostic value for LBLP. To establish if and what kind of muscle atrophy is indicative of radicular or non-radicular pain, further studies concerning the size of MV decrease combined with the results of clinical examinations are required.
